# Analysis of the complete mitochondrial genome of click beetle *Agriotes hirayamai* (Coleoptera: Elateridae)

**DOI:** 10.1080/23802359.2018.1443041

**Published:** 2018-02-26

**Authors:** Aili Lin, Xincheng Zhao, Nan Song, Te Zhao

**Affiliations:** College of Plant Protection, Henan Agricultural University, Zhengzhou, People’s Republic of China

**Keywords:** *Agriotes hirayamai*, mitochondrial genome, phylogenetic analysis

## Abstract

In this study, the complete mitochondrial genome sequence of click beetle *Agriotes hirayamai* (GenBank accession no. MG728108) was obtained using next-generation sequencing (NGS) method. The complete mitochondrial genome of *A. hirayamai* is 16,156 bp in length and contains 13 protein-coding genes, 22 transfer RNAs, two ribosomal RNAs and a control region. The gene arrangement is consistent with the typical insect mitochondrial genome. Maximum likelihood tree shows that the newly sequenced *A. hirayamai* cluster with other two sampled species of *Agriotes* and the family Elateridae is monophyletic.

The family Elateridae, commonly known as click beetles due to a unique and well-known startling defence mechanism named ‘clicking’ is the largest family of Elateroidea and contains 9000 species classified to about 400 genera (Lawrence 1982). Here, we determined the complete mitochondrial genome of *Agriotes hirayamai*, which is the second mitochondrial genome sequenced to date in the genus of *Agriotes.*

The sampled specimen was collected from the city of Xinyang, China (the geospatial coordinates: 114.083°E, 31.833°N). The specimen was stored in the Entomological Museum of Henan Agricultural University (voucher no. MT-Zz15071207). We extracted the total genomic DNA from muscular tissue preserved in the absolute ethyl alcohol at −20 °C using the TIANamp Micro DNA Kit (Tiangen Biotech Co., Ltd., Zhongguancun, Beijing, China). We constructed the library composed of the genomic DNA of *A. hirayamai* and of other insects unrelated to this study using Illumina HiSeq 2500 platform (PE 150). The raw reads were *de novo* assembled by the SOAPdenovo software (Zhao et al. 2011), with an average 358.98 × coverage. We identified the complete mitochondrial genome of *A. hirayamai* from a single large contig (16,245 bp), by blasting the pre-determined mitochondrial *cox1*, *cytb* and *rrnS* gene fragments against the assembled contigs with BioEdit7.0.9.0 (Hall 1999).

The complete mitochondrial genome of *A. hirayamai* (GenBank accession no. MG728108) is 16,156 bp in length after removing the overlapping regions. The full mitochondrial genome contains 13 protein-coding genes (PCGs), 22 transfer RNAs (tRNAs), two ribosomal RNAs (rRNAs) and a putative control region (CR). The gene arrangement of *A. hirayamai* is found to be similar to most insect mitochondrial genomes (Wolstenholme 1992). All PCGs of *A. hirayamai* start with the typical start codon ATN except for *nad1*, which begins with the putative start codon TTG. Nine PCGs of *A. hirayamai* use TAA or TAG as stop codon, but four of the genes (i.e. *cox2*, *cox3*, *nad4* and *nad5*) use T or TA as the incomplete stop codon. All 22 tRNA genes can be folded into the typical cloverleaf structure except for *trnS1*, in which the dihydrouracil arm cannot form a stable stem-loop structure but a simple loop. And the anticodon of *trnS1* is UCU rather than usual GCU. The CR is 1490 bp in length, which consists of A + T content of 84.09% and G + C content of 15.91%.

Maximum likelihood tree recovered the monophyletic *Agriotes* with strong bootstrap support, which was comprised of the newly sequenced *A. hirayamai* and other two representatives of *Agriotes* (Figure 1). The family Elateridae was found to be monophyletic (BP = 91), which was consistent with the previous studies (Douglas 2011; Kundrata and Bocak 2011).

**Figure 1.**
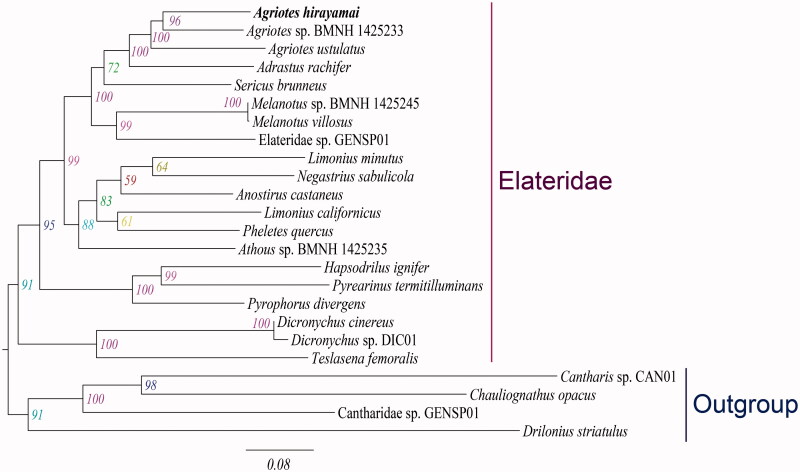
Maximum-likelihood tree inferred from the mitochondrial genome data. The maximum-likelihood analysis was reconstructed by the concatenated nucleotide sequences of 13 mitochondrial protein-coding genes (11,109 bp) using IQ-TREE (Nguyen et al. 2015). Numbers alongside nodes refer to bootstrap support values. The newly determined species is indicated in bold. All GenBank accession numbers for species included in this study are listed as following: *Adrastus rachifer* (KX087232), *Agriotes hirayamai* (MG728108), *Agriotes* sp. BMNH 1425233 (KT876879), *A. ustulatus* (JX412737), *Anostirus castaneus* (KX087237), *Athous* sp. BMNH 1425235 (KT876881), Cantharidae sp. GENSP01 (JX412853), *Cantharis* sp. CAN01 (JX412749), *Chauliognathus opacus* (FJ613418), *Dicronychus cinereus* (KX087283), *Dicronychus* sp. DIC01 (JX412848), *Drilonius striatulus* (JX412822), Elateridae sp. GENSP01 (JX412817), *Hapsodrilus ignifer* (KJ922149), *Limonius californicus* (KT852377), *L. minutus* (KX087306), *Melanotus* sp. BMNH 1425245 (KT876904), *Melanotus villosus* (KX087314), *Negastrius sabulicola* (KX087320), *Pheletes quercus* (KX087332), *Pyrearinus termitilluminans* (KJ922150), *Pyrophorus divergens* (EF398270), *Sericus brunneus* (KX087344), *Teslasena femoralis* (KJ938491).
